# Genome Sequences of Three *Helicobacter pylori* Strains from Patients with Gastric Mucosa-Associated Lymphoid Tissue Lymphoma

**DOI:** 10.1128/genomeA.00229-15

**Published:** 2015-04-23

**Authors:** Hsuan-Chen Wang, Feng-Chi Cheng, Ming-Shiang Wu, Hung-Yu Shu, H. Sunny Sun, Yu-Chun Wang, Ih-Jen Su, Chi-Jung Wu

**Affiliations:** a National Institute of Infectious Diseases and Vaccinology, National Health Research Institutes, Miaoli, Taiwan; b Institute of Population Health Sciences, National Health Research Institutes, Miaoli, Taiwan; c Department of Internal Medicine, National Taiwan University Hospital, Taipei, Taiwan; d Department of Bioscience Technology, Chang Jung Christian University, Tainan, Taiwan; e Institute of Molecular Medicine, College of Medicine, National Cheng Kung University, Tainan, Taiwan; f Department of Internal Medicine, National Cheng Kung University Hospital, Tainan, Taiwan

## Abstract

Most of the published complete genome sequences of *Helicobacter pylori* strains are limited to clinical isolates associated with gastritis, peptic ulcers, or gastric cancer. The genome sequences of three *H. pylori* strains isolated from patients with gastric mucosa-associated lymphoid tissue (MALT) lymphoma are presented here to facilitate studies of *H. pylori*-associated MALT lymphomagenesis.

## GENOME ANNOUNCEMENT

*Helicobacter pylori*, a Gram-negative microaerophilic bacterium, has been implicated in a variety of human gastroduodenal diseases, including chronic gastritis, peptic ulcers, and gastric cancer (1). *H. pylori* also plays an oncogenic role in the development of mucosa-associated lymphoid tissue (MALT) lymphoma. Chronic immunological stimulation by *H. pylori* leads to the formation of acquired MALT in the gastric mucosa. These infiltrating B cells actively proliferate and occasionally undergo malignant transformation (2). While several virulence factors of *H. pylori* that are involved in the pathogenic processes of peptic ulcers and gastric cancer have been described, such as CagA and VacA, the virulence determinants associated with MALT lymphomagenesis remain undetermined (3, 4). Furthermore, with the exception of *H. pylori* B38, a gastric MALT lymphoma strain, most of the published complete genome sequences of *H. pylori* strains are limited to clinical isolates associated with gastritis, peptic ulcers, gastric cancer, or asymptomatic colonization (4). In the present report, the genome sequences of three *H. pylori* strains, HPML01, HPML02, and HPML03, which were isolated from three patients with gastric MALT lymphoma, were analyzed to facilitate studies on *H. pylori*-associated MALT lymphomagenesis.

Whole-genome sequencing of HPML01, HPML02, and HPML03 was performed using 454 sequencing technology. Genomic shotgun and 8-kb paired-end libraries were constructed and sequenced with a 454-GS Junior instrument (Roche Diagnostics, Indianapolis, IN). The sequence reads of the three strains were assembled into 33, 55, and 45 contigs and 4, 2, and 4 scaffolds, respectively, using 454 Newbler (version 2.7; 454 Life Sciences, Branford, CT). All gaps between the contigs were filled by PCR and Sanger sequencing, and the complete genome sequences were assembled and analyzed using OMIGA 2.0 and the CG-Pipeline software (5). The final draft genomes consisted of circular chromosomes of 1,629,815 bp, 1,562,125 bp, and 1,629,114 bp in length for HPML01, HPML02, and HPML03, respectively, as well as a circular plasmid of 6,220 bp for HPML03. The pertinent statistics of the genomes are summarized in Table 1.

**TABLE 1:** Summary of statistics for three sequenced gastric MALT lymphoma *H. pylori* strains

Strain	Genome content	Accession no.	Genome length (bp)^a^	Coverage depth (fold)^b^	G+C content (%)^a^	No. of predicted genes^c^	No. of coding sequences^c^
HPML01	Circular chromosome	AP014710	1,629,815	67	38.69	1,707	1,671
HPML02	Circular chromosome	AP014711	1,562,125	45	38.92	1,751	1,715
HPML03	Circular chromosome and circular plasmid	AP014712 (chromosome) and AP014713 (plasmid)	1,629,114 and 6,220	60	38.67	1,744	1,708

a Determined by OMIGA.

b Determined by 454 Newbler.

c Determined by CG-Pipeline software.

The genome sequences were annotated using the NCBI Basic Local Alignment Search Tool. HPLM01, HPLM02, and HPLM03 harbored genes encoding previously known virulence factors associated with peptic ulcers and gastric cancer, such as *cagA*, *cagE*, *vacA*, *iceA*, *babA*, *hopQ*, *oipA*, *sabA*, *hopZ*, *babB*, *babC*, *sabB*, and *homB*, while the last four genes were not present in strain B38 (3, 4). A whole-genome comparison using the OrthoMCL software (6) revealed that there are 9 genes shared by HPLM01, HPLM02, and HPLM03 that are absent in the five published *H. pylori* strains (namely, 26695 [recovered from a patient with gastritis], HP J99 [duodenal ulcer], HPAG1 [atrophic gastritis], G27 [unspecified], and Shi470 [chronic gastritis]) (7–11), eight of which are hypothetical proteins. HPLM01, HPLM01, and HPLM03 also were found to share many gene substitution, deletion, and insertion sites that are not present in the five published strains. Although the biological significance of this genetic variability needs to be functionally validated, knowledge of the genome sequences of MALT lymphoma *H. pylori* strains opens new avenues for the further genomics-based exploration of virulence determinants contributing to MALT lymphomagenesis.

### Nucleotide sequence accession numbers. 

The genome sequences of HPLM01, HPLM02, and HPLM03 were deposited into the whole-genome sequencing (WGS) database of DDBJ/EMBL/GenBank under the accession numbers listed in Table 1. The versions described in this paper are the first versions.
